# A fresh look at paralytics in the critically ill: real promise and real concern

**DOI:** 10.1186/2110-5820-2-43

**Published:** 2012-10-12

**Authors:** David Price, Nicholas J Kenyon, Nicholas Stollenwerk

**Affiliations:** 1 Division of Pulmonary, Critical Care and Sleep Medicine, Univ. of California, Davis, 4150V. Street, Suite 3400, Sacramento, CA 95817, USA; 2 Department of Internal Medicine, University of California, Davis, USA

**Keywords:** Neuromuscular blocking agents, Neuromuscular nondepolarizing agents, Polyneuropathies, Respiratory distress syndrome, Adult, Cisatracurium, Status asthmaticus, Shock, Septic

## Abstract

Neuromuscular blocking agents (NMBAs), or “paralytics,” often are deployed in the sickest patients in the intensive care unit (ICU) when usual care fails. Despite the publication of guidelines on the use of NMBAs in the ICU in 2002, clinicians have needed more direction to determine which patients would benefit from NMBAs and which patients would be harmed. Recently, new evidence has shown that paralytics hold more promise when used in carefully selected lung injury patients for brief periods of time. When used in early acute respiratory distress syndrome (ARDS), NMBAs assist to establish a lung protective strategy, which leads to improved oxygenation, decreased pulmonary and systemic inflammation, and potentially improved mortality. It also is increasingly recognized that NMBAs can cause harm, particularly critical illness polyneuromyopathy (CIPM), when used for prolonged periods or in septic shock. In this review, we address several practical considerations for clinicians who use NMBAs in their practice. Ultimately, we conclude that NMBAs should be considered a lung protective adjuvant in early ARDS and that clinicians should consider using an alternative NMBA to the aminosteroids in septic shock with less severe lung injury pending further studies.

## Introduction

Since the publication of the 2002 guidelines for the use of neuromuscular blocking agents (NMBAs) in the critically ill, titled “Clinical practice guidelines for sustained neuromuscular blockade in the adult critically ill patient” [1], there has been new evidence that clinicians must reconcile with their current understanding of which patients should receive NMBAs. Whereas NMBAs are used in the intensive care unit (ICU) for a variety of indications, in this review we focus on the use of NMBAs to facilitate mechanical ventilation, especially in acute respiratory distress syndrome (ARDS). This review begins by contrasting the 2002 guidelines with current practice patterns related to NMBAs. In the “Promising Uses” section, we present the newest evidence to support the use of NMBAs in early ARDS with a focus on possible mechanisms for these results. We ultimately suggest clinicians to consider NMBAs as part of a lung protective strategy, especially in patients with more severe lung injury. In the section titled “Cause for Concern,” we evaluate the evidence linking NMBAs to CIPM in different disease states. Specifically, we use human as well as preclinical studies to raise concern about the routine use of NMBAs, especially aminosteroid NMBAs, in septic shock. Finally, we synthesize this data for clinicians by offering a suggested alternative algorithm to the one presented in the 2002 guidelines.

### Clinical pharmacology of NMBAs

Neuromuscular blocking agents act on the skeletal muscle postsynaptic nicotinic acetylcholine (ACh) receptor. This class of medications is broken down into depolarizing and nondepolarizing blockers. Depolarizing NMBAs, the prototype being succinylcholine, are rarely used in critically ill patients because of the risk of hyperkalemia and malignant hyperthermia and will not be further addressed in this review. Nondepolarizing NMBAs competitively bind the alpha subunits of the intra-junctional ACh receptor on the skeletal muscle postsynaptic membrane leading to inhibition of current through the receptor and thus flaccidity [2]. The clinical pharmacology of these agents is presented in Table 1.

**Table 1:** Clinical pharmacology of nondepolarizing neuromuscular blocking agents

**NMBA**	**Peak effect (min)**	**Recovery (min)**	**Metabolism**	**Renal elimination (%)**	**Biliary elimination (%)**	**Vagolytic effect**	**Histamine release**	**Critical illness polyneuromyopathy**
**Benzylisoquinolinium**								
Atracurium	2-3	30-60	Hoffman Elimination (blood)	5-10	None	None	+	+
Cisatracurium	1-7	40-90		None	None	None	None	+
**Aminosteroid**								
Pancuronium	2-3	80-180	Liver	40-70	10-15	+++	None	+++
Rocuronium	1-2	20-60		10-30	50-75	+	None	+
Vecuroniurn	2-3	40-60		15-50	35-50	None	None	+++

+ minimal, ++ moderate, +++ marked.

### Pattern of NMBA use

The 2002 guidelines identify indications for NMBAs and offer a simple algorithm for selecting an agent [1]. Recent surveys and cohort studies of clinical practice show a more detailed picture of actual NMBA use. Whereas the agents used and the indications for use are similar to the guidelines, these studies show a practice pattern of NMBAs used disproportionately on the sickest patients.

Pancuronium, rocuronium, and vecuronium are the most commonly used NBMAs [3,4]. In their survey of U.S. intensivists, Rhoney and Murry [3] found 50% of respondents use vecuronium frequently or routinely versus 25% who use pancuronium and 6.4% who use rocuronium. When asked about decision-making, clinicians responded that they were more likely to choose a NMBA based on their clinical experience and preference then on patient-specific factors. The surveys found that surgical ICU patient are more likely to receive pancuronium, whereas medical ICU patients are more likely to receive vecuronium. As for administration, clinicians are more likely to give vecuronium and cisatracurium as continuous infusions, whereas all other agents are mostly given as intermittent boluses [3].

**Figure 1:**
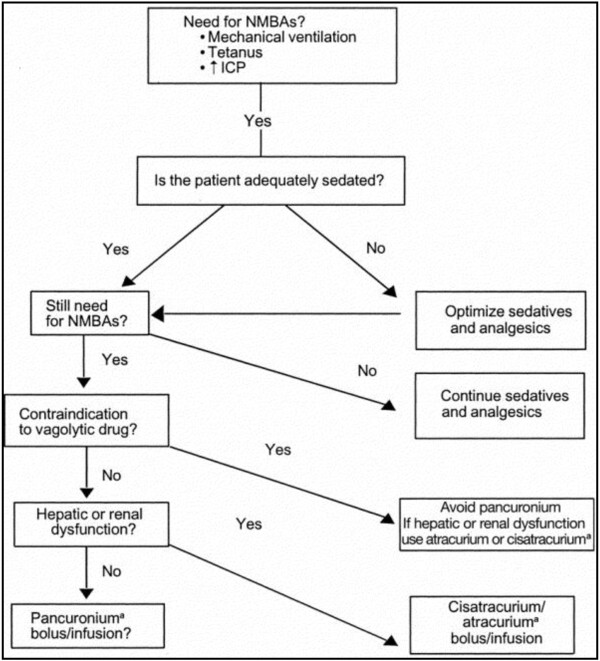
**Clinical practice guidelines for sustained neuromuscular blockade in the adult critically ill patient. **Reprint with permission from [1].

The indications reported by Rhoney and Murry [3] and Mehta et al. [4] for NMBA use are consistent with the 2002 guidelines (Figure 1). Beyond endotracheal intubation, the facilitation of mechanical ventilation is the most common indication for NMBAs with half reporting routine or frequent use of NMBAs for this reason. Less commonly cited indications include dosing to decrease metabolic demand, control of intracranial pressure, and decrease agitation. Within the category of facilitating mechanical ventilation, the most cited reasons included use of unconventional ventilation (35%), hypoxemia (25%), reduced compliance (25%), patient ventilator asynchrony (18%), and hypercapnia (15%) [4].

Arroliga et al. [5] went beyond surveys and looked at a cohort of international patients to determine which patient’s received NMBAs; 13% of patients in this cohort received a NMBA. Use was associated with patients on full ventilatory support with an odds ratio (OR) of 3.68 and 95% confidence interval (CI) of 2.38-5.7, requiring permissive hypercapnia (OR 4.49, CI 2.53–7.95), prone ventilation (OR 4.36, CI 2.33–8.12), with higher pressures, higher positive end-expiratory pressure (PEEP), and patients with ARDS (OR 2.01, CI 1.43–2.83). A subsequent study by Arroliga et al. [6] focused on only ARDS patients and similarly showed use associated with sicker patients. Intensity of NMBA use in this population was associated with higher acute physiology and chronic health evaluation III (APACHE III) score, higher plateau pressures, larger Alveolar-arterial oxygen concentration (A-a) gradient, and lower tidal volumes.

Despite firm recommendation that NMBA effect should be monitored in the ICU [1], in practice, the use of regular monitoring and daily NMBA interruption protocols remains variable. Depending on the study, 25-50% of intensivists report protocols for NMBA use in their ICUs. The rate of monitoring with train of four (TOF) is better with up to 84% using electrical monitoring [3,4].

Finally, the duration of NMBA use is variable but favors shorter duration of use. Arroliga et al.’s international study [5] of mechanically ventilated patients reported median duration of use to be 2 days. In acute lung injury and ARDS patients, however, NMBAs are used for even shorter periods [6].

### Promising uses: ARDS

There is growing evidence to support the selective use of NMBAs in early ARDS. In the past decade, three prospective randomized trials have evaluated the use of NMBAs in ARDS [7-9]. Before these trials, NMBAs were thought to be beneficial in ARDS [2,10], but there was insufficient evidence to support a mechanism or demonstrate a benefit.

### Improve oxygenation

Gainnier et al. [7] randomized 56 medical and surgical ICU patients with ARDS in four French hospitals to 48 hours of cisatracurium or placebo to evaluate the primary endpoint of improved oxygenation within 120 hours of randomization. Statistically significant improvement in oxygenation was noted in the experimental group within 48 hours and was sustained through the primary endpoint of 120 hours (*p* = 0.021). Of note, Gainnier’s experimental group abolished all TOF responses; deeper paralysis than is called for by the guidelines [1]. Forel et al*.*[8] found a similar improvement in oxygenation as a secondary endpoint. The time to effect in these studies differ from Coggeshall’s [10] observation of immediate improvement in oxygenation after administration of pancuronium to a patient with severe ARDS.

The hypothetical mechanism for this sustained improvement in oxygenation is likely a combination of reduced oxygen consumption [11,12] as well as homogenous distribution of PEEP and tidal volume limiting disproportionate barotrauma [9] and worsening of ARDS. These effects appear to occur in the absence of improved pulmonary mechanics [13,14]. Reduced oxygen consumption was previously postulated as a mechanism for improved oxygenation in a severe ARDS patient with an arterial partial pressure of oxygen to inspired oxygen (P:F) ratio of 58 and elevated respiratory rate [10]. With each dose of NMBA, the patient’s respiratory rate decreased and oxygenation improved. This effect was later quantified in eight healthy adults whose oxygen consumption decreased 18% as they were transitioned from continuous positive airway pressure (CPAP) alone to invasive ventilation with a NMBA [11]. Reduced oxygen consumption is an immediate effect, however, whereas the improvements in oxygenation elsewhere reported were seen after 24 hours [7,8,15]. This delayed improvement in oxygenation is better explained by slowing of disease progression. Papazian et al. [9] found decreased barotrauma in patients receiving NMBAs as opposed to the original lung protective study [15], whereas despite lower tidal volume in the experimental group, the amount of barotrauma was similar in each group. This decreased stress likely potentiates the lung protective effect of low tidal volume, because it leads to more homogenous distribution of ventilation [16].

### Decrease inflammatory response

One of the more exciting findings is that NMBAs may decrease the inflammatory response associated with ARDS. Forel et al. [8] demonstrated this result in a 2006 study, which randomized 36 patient to 48 hours of NMBA versus placebo and compared their bronchoalveolar lavage (BAL) and serum at 0 and 48 hours for statistical differences in inflammatory markers. Cisatracurium was again administered to the experimental group to abolish train of four responses. At 48 hours, there was a statistically significant decrease in interleukin-1 (IL-1), IL-6, and IL-8 in the BAL and IL-1 and IL-6 in the serum. No difference was found in the BAL or serum level of tumor necrosis factor-alpha or the BAL cell differential.

Although the mechanism is unknown, it is possible that NMBAs eliminate excessive intrathoracic pressure changes and decrease alveolar overdistention, a phenomenon that is known to release proinflammatory cytokines in animal models [17-21]. IL-6 levels measured in the original lung protective trial [15] were significantly lower in the lung protective group. This decrease in inflammatory cytokines with lung protective tidal volumes was previously studied [22,23], with Parson et al. showing mortality benefit with decreased IL-6 levels. With the administration of a NMBA and the decreased mechanical stress and improved homogenation discussed above, there is less inflammatory response and less likelihood of this inflammation becoming systemic.

### Improve mortality

In 2010, Papazian et al. [9] was the first to show a mortality benefit with early use of NMBAs in ARDS. This result was in contrast to a prior retrospective cohort study [6] that showed no mortality benefit. Building on the work by Gainnier and Forel, Papazian randomized 340 patients to 48 hours of cisatracurium versus placebo and was able to show a 90-day mortality benefit after adjusting for baseline P:F ratio, simplified acute physiology score II (SAPS II), and plateau pressure (hazard ratio 0.68, CI 0.48–0.98, *p* = 0.04). There was not, however, a significant difference in crude mortality between the study groups (31.6% in the cisatracurium group vs. 40.7% in the control group, *p* = 0.08). Beyond the improved oxygenation and anti-inflammatory mechanism mentioned above, the etiology of this adjusted mortality benefit from NMBAs can be inferred from the subgroup analysis. Patients in the cisatracurium group had more ventilator-free days (53 vs. 45, *p* = 0.03), more days outside the ICU (48 vs. 40, *p* = 0.03), less barotrauma, as well as more days without coagulation abnormalities, renal failure, and hepatic dysfunction. The greatest benefit, moreover, was seen with a P:F ratio less than 120. Given the weakly significant *p* value associated with adjusted mortality, it is worth noting that this study was underpowered for the mortality rate it ultimately saw in its control group.

### Cause for concern: critical illness polyneuromyopathy

Critical illness polyneuromyopathy (CIPM) is a term that describes two separate diseases: critical illness polyneuropathy (CIP) and critical illness myopathy (CIM). Whereas CIP mainly affects motor and sensory nerve fibers leading to degeneration of the skeletal muscle, CIM directly affects myosin leading to muscle necrosis. In the critically ill, CIP portends a worse prognosis than CIM [24]. CIP has been associated with increased in-hospital mortality [25], longer duration of mechanical ventilation [26,27], increased days in the ICU [28], and longer hospital stays [26]. In practice, CIM and CIP can overlap and often are indistinguishable without electromyographic (EMG) studies and muscle biopsy. For the purpose of this review, CIPM will be used to describe clinically significant weakness that is not further defined, whereas CIP and CIM will be used when authors provide sufficient EMG, muscle biopsy, and clinic data on sensory involvement to make a distinction.

CIPM has haunted physicians using NMBAs since this debilitating side effect was described in severe asthmatic patients who were receiving both high-dose steroids and NMBAs [26,27,29]. Despite legitimate concerns with prolonged use of NMBAs, there is little evidence that using NMBAs for less than 48 hours causes CIPM. The relationship between NMBAs and CIPM is not that simple, however, but a number of animal and human studies have helped to illuminate the connection and alert clinicians about which patients are at highest risk for CIPM.

### CIPM with Benzylisoquinolinium versus Aminosteroid NMBAs

As seen in Table 2, aminosteroid NMBAs have been more strongly implicated in CIPM than the benzylisoquinolinium compounds. Aminosteroid NMBAs have been associated with CIPM in patients receiving corticosteroids [26,27,30], sepsis with multiorgan failure [25,31], in multiple animal models of NMBA use [32,33], and with both a dose- and time-dependent relationship [27,31,34]. Conversely, only case studies of benzylisoquinolinium agents with concomitant corticosteroids for at least 6 days have linked this class of NMBAs to CIPM [35-38]; a link that could be explained by use of corticosteroids alone. In their studies of 48 hours of cisatracurium in ARDS, moreover, Papazian, Gainnier, and Forel found no association between cisatracurium and CIPM.

**Table 2:** Studies of NMBAs related to prolonged weakness, CIP, and CIM

**NMBA**	**Author**	**Subjects**	**Study design**	**Motor**	**Sensory**	**EMG**	**Muscle biopsy**	**Prolonged weakness, CIP, and CIM**
**Benzylisoquinolinium**								
Cisatracurium								
*Human studies*								
	Fodale et al. [36]	1	Case report	Y	N	Y	N	NMBA and CS for 7 days in patient with chest wall trauma led to quadriplegia consistent with CIM.
	Davis et al. [35]	1	Case report	Y	N	N	N	NMBA and CS for 6 days with 6 additional days of NMBA in 45-year-old with ARDS led to CIM.
Atracurium								
*Human studies*								
	Tousignant et. al. [38]	1	Case report	Y	N	Y	N	18-year-old asthmatic with 7 days of NMBA and CS develops acute quadriparesis 3 days after cessation of NMBA consistent with CIM.
	Meyer et al. [37]	2	Case report	Y	N	Y	N	38-year-old receiving CS and 8 days of NMBA developed CIM. 25-year-old with good pastures receiving CS and NMBA for 6 days develop CIM.
**Aminosteriods**								
Pancuronium								
*Human studies*								
	Behbehani et al. [26]	86	Retrospective Cohort	Y	N	Y	N	Asthmatics receiving NMBA and CS. Pancuronium, vecuronium used in 30 with 9 developing CIM. All nine received pancuronium.
	de Lemos et al. [39]	30	Prospective Observational Cohort	Y	N	Y	N	Study of recovery time in continuous infusion versus bolus groups. Six patients with CIM, five of which received continuous infusion. No statistical difference in total dose between groups.
	Giostra et al. [30]	9	Prospective Cohort	Y	Y	Y	Y	Over 2 years, nine patients with respiratory failure requiring mechanical ventilation and NMBA developed CIP. Eight of nine received concomitant NMBA and CS.
Rocuronium								
*Animal Studies*								
	Maes *et al.*[40]	Rat / 27	Prospective Randomize	N/A	N/A	Y	Y	1 dose of CS added to 24 hours of NMBA results in decreased CIM of diaphragm than NMBA alone.
	Testelmans *et al.*[33]	Rat / 24	Prospective Randomize	N/A	N/A	Y	Y	24 hours of rocuronium associated with worse CIM than cisatracurium.
	Testelmans *et al.*[32]	Rat / 34	Prospective Randomize	N/A	N/A	Y	Y	24 hours of NMBA associated with increased CIM than mechanical ventilation alone.
Vecuronium								
*Human Studies*								
	Garnacho- Montero *et al.*[25]	73	Prospective Cohort	Y	Y	Y	N	In septic cohort with more than 2 organ failure, 9 of 10 patients who got NMBA developed CIP. 6 received vecuronium and 3 received atracurium
	Rudis *et al.*[34]	77	Prospective Randomized Single-Blind	Y	N	Y	N	Use of peripheral nerve stimulator resulted in half dose of NMBA given. 16 patients with prolonged blockade and 4 with CIP. More prolonged blockade and CIP in group with more NMBA.
	Prielipp *et al.*[41]	58	Prospective Randomized Double-Blind	Y	Y	Y	Y	Prolonged recovery in 13 patients with vecuronuim versus 2 with cisatracurium (p=0.002). CIP in 1 vecuronuim patient.
	Douglass *et al.*[27]	25	Prospective Cohort	Y	N	N	N	22 patients received NMBA and CS. 9 developed CIM. CIM associated with time ventilated and dose of NMBA received.
	Kupfer *et al.*[31]	28	Prospective Cohort	Y	1/5	Y	N	50% of patient without sepsis or multi-organ failure with more than 6 hours of NMBA infusion developed weakness. 1 CIPM, 4 CIM. CIPM and CIM ssociate with increase dose.
	Danon *et al.*[42]	1	Case report	Y	N	N	Y	20 year old asthmatic who received CS and NMBA for 10 days developed CIM.

Y, yes; N, no; N/O, not obtained; CS, corticosteroid; CIP, critical illness polyneuropathy; CIM, critical illness myopathy; CIPM, critical illness polyneuromyopathy; NMBA, neuromuscular blocking agent; ARDS, acute respiratory distress syndrome [25-27,30-38,44-48].

### CIPM with NMBAs in general population

In a general population of ICU patients, de Jonghe et al. [44] found a CIPM incidence of 25% but no association between NMBAs and CIPM. Rather, CIPM was associated with corticosteroid administration, duration of mechanical ventilation, and number of days with dysfunction in more than two organs. Beyond this study, however, within subgroups of patients receiving NMBAs, specifically patients with ARDS, severe asthma on steroids, and septic shock, the association with CIPM is more variable.

### CIPM with NMBAs in ARDS

Papazian, Gainnier, and Forel all used NMBAs for 48 hours in patients with either ALI or ARDS and found no association between NMBAs and CIPM. In the Papazian study, at 28 days, the difference in patients without ICU-acquired paresis was not statistically significant between the cisatracurium and control group (*p* = 0.64). No studies have evaluated incidence of CIPM in ARDS when NMBAs are used for more than 48 hours.

### CIPM with NMBAs in severe asthmatics receiving corticosteroids

The concomitant use of NMBAs and corticosteroids has been shown to place patients at high risk for CIM. If clinically necessary, however, limiting the duration of using these medications together can minimize CIM. The first two cohort studies to evaluate CIM in severe asthmatics receiving corticosteroids and NMBAs, Douglass et al. and Leatherman et al. [27,29], found the incidence of CIM to be roughly one third. When time paralyzed is factored in, however, these numbers tell a different story. In the Douglass study, the patients with CIM were paralyzed for an average of 5 days compared with an average of 1 day in the CIM-free group. Moreover, in the Leatherman study, when CIM group was broken down by time paralyzed, 6% of patients paralyzed less than 24 hours developed CIM versus 38% paralyzed for 24 to 48 hours and 85% paralyzed for more than 48 hours. In neither of these studies was incidence of CIM adjusted for severity of underlying illness. In contrast, when Behbehani et al. [26] analyzed a cohort of severe asthmatics patients receiving corticosteroids and adjusted for severity of underlying illness, they found no association between use of NMBAs and CIM. They did, however, find an association between duration of muscle paralysis and development of CIM with an odds ratio of 2.1 per day of NMBA used.

### CIPM with NMBAs in septic shock

The evidence linking the routine use of NMBAs in septic shock with the development of CIP and CIPM is concerning. Sepsis itself is a risk factor for CIPM with rates reported as high as 70% [28]. Garnacho-Montero et al. [25] specifically looked at a cohort of septic patients with greater than two MODS who required mechanical ventilation for more than 10 days to evaluate the impact of CIP on outcomes. The incidence of CIP was 63% at day 10. Among the ten septic patients to receive NMBAs, however, the incidence of CIP was 90%. Six of the nine patients with CIP received vecuronium, whereas the other three received atracurium. The odds ratio for risk of CIP with use of NMBAs in their cohort was 16 (OR 16.32; 95% CI 1.34-199; *p* = 0.0008). Of note, the one patient without CIP received less total NMBA than the three patients with CIP. In Forel’s study of NMBAs in ARDS [8], moreover, 2 of 36 patients developed CIP: 1 who received cisatracurium and the other who did not. Both of these patients had ARDS from septic shock. Sepsis appears to be such a potent risk factor for CIPM that use of an additional agent with potential to cause CIPM, such as NMBAs, places patients at substantial risk for CIPM.

Animal model studies support this clinical finding of sepsis as a potent contributor to CIPM. Ochala et al. [45] intubated, sedated, and induced sepsis through *E. coli* endotoxin in young piglets for 5 days to evaluate the different contribution that mechanical ventilation, corticosteroids, NMBAs, and sepsis play in the pathogenesis of CIM. Whereas mechanical ventilation was consistently shown to decrease muscle compound muscle action potential amplitudes (CMAP), sepsis was independently associated with a dramatic decrease in force generation capacity, a finding not seen with NMBAs. It should be noted that when all interventions were involved (intubated and sedated, septic, corticosteroids, and NMBA), importantly, the degree of decline in CMAP was greatest. This finding supports the idea that NMBAs administered in the setting of sepsis are more likely to cause CIPM than sepsis alone. Rossignol et al. [46] found similar findings in septic rats that were not intubated. After 10 days of sepsis, the fast-twitch extensor digitorum longus muscle has a smaller cross-sectional area, increased fatigability, and reduction in maximal twitch contraction among other abnormalities.

Given the concerning evidence linking septic shock and NMBAs with CIPM presented above, we believe that clinicians should use caution when considering NMBAs in this patient population. Moreover, the strong association between aminosteroid NMBAs and CIPM described above and shown in Table 2 should further raise concern about using aminosteroid NMBAs in these patients already at high risk for CIPM.

### Sedation and monitoring of NMBAs

Before the initiation of NMBAs, clinicians must ensure appropriate sedation and patient comfort. Practically, clinicians can provide sedation and analgesic until the patient is unconscious before administration of an NMBA [1]. Once properly sedated, accurate monitoring of NMBAs allows clinicians to get the maximum benefit while limiting the amount of medication used. Increased dose and duration of NMBA is associated with significant morbidity [25-27,29], so it is in the patient’s best interest to given the least amount of NMBA possible. Secondary to underlying organ dysfunction and intrinsic characteristics, patients require different amounts of NMBA to achieve the same level of paralysis. For example, Circeo et al. [47] found that patients with multiorgan failure (MOF) required less than half the dose of NMBA than patients without MOF to achieve same level of paralysis. In a study of septic rats, conversely, atracurium has a shorter onset and wore off quicker than in nonseptic rats [46].

The depth of paralysis, however, remains controversial. Targeting a TOF of two of four rather than zero of four has been shown to be beneficial. In a prospective, randomize, open-labeled study [48] of 102 ARDS patients randomized to shallow or deep paralysis for a median duration of 31.4 (range 1.6-650.6) and 28.9 (range 3.1-219.7) hours respectively, the shallow paralysis group had a higher P:F ratio, lower plateau pressure, and perhaps most importantly, received less total NMBA and had shorter recover time. Moreover, the 2002 guidelines, as discussed above, also recommend targeting a TOF of one to two of four. Conversely, in their studies that showed improved oxygenation, inflammation, and mortality with NMBAs in early ARDS, Gainnier, Forel, and Papazian all targeted zero of four.

### Areas for future study

This paper brings to light multiple clinical questions that require further experimentation to clarify. To begin with, could the improvements in oxygenation, inflammation, and mortality shown in studies of NMBAs in ARDS by Gainnier, Forel, and Papazian been achieved with less paralytic? Although there is some evidence [47,48] as well as guidelines [1] available that targeting a TOF of two of four is beneficial, these authors chose a greater depth of paralysis. Perhaps the small mortality benefit shown by Papazian would have been larger with less paralytic. Moreover, we offered evidence to support a mechanism for the improvement in oxygenation and inflammation seen in the studies by Gainnier and Forel, respectively, but additional studies are needed to investigate these hypothetical mechanisms.

More studies are needed to address the use of NMBAs in patients with sepsis, and more specifically, septic shock. The animal studies and the few human studies discussed here suggest an unacceptable level of CIPM when NMBAs are used in septic shock, especially aminosteroid NMBAs. Further, prospective, randomized studies that evaluate aminosteroid NMBAs versus benzylisoquinolinium NMBAs in septic shock and the association with CIPM are needed to bolster our concern.

## Conclusions

Achieving a mortality benefit from use of NMBAs comes with a small margin for error. Future studies of these agents need to build on the body of evidence suggesting that NMBAs can improve outcomes in patients with lung injury. The following conclusions should be kept in mind.

**Figure 2:**
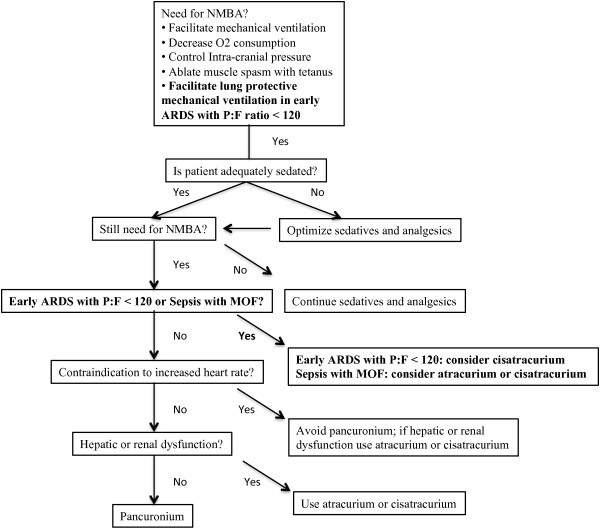
**Suggested modifications to clinical practice guidelines for sustained neuromuscular blockade in the adult critically ill patient. **Bolded text represents suggested modifications to 2002 guidelines.

1. Use of NMBAs should be targeted. NMBAs offer a benefit in ARDS, whereas there is evidence that they may cause harm in septic shock. Within the broad category of ARDS, patients with a P:F ratio less than 120 have the greatest mortality benefit. To help clinicians select the proper NMBA, in Figure 2 we offer an algorithm for selecting a NMBA, which builds on the 2002 guidelines by incorporating the evidence presented in this review.

2. The less NMBA used the better. This conclusion applies more to time paralyzed than to depth of paralysis. The use of NMBAs for less than 48 hours is supported by more evidence than any other conclusion related to NMBAs. There is evidence, moreover, that less than 48 hours of NMBAs, either 24–36 hour may be even more beneficial.

3. Beware of potentiation of effect. Whereas corticosteroids and aminoglycosides are known to potentiate CIPM, of equal importance is presence of sepsis with MOF. Clinicians must balance the risk-benefit ratio based on a complete understanding of the patient’s medications and clinical course. Extrapolating the findings of recent positive studies in ARDS to all lung injury patients requiring mechanical ventilation is difficult, but the answer to this challenge represents the future of “paralytics” in the ICU.

## Endnotes

No endnotes included in this review.

## Abbreviations

A-a gradient: Alveolar-arterial oxygen concentration gradient; ACh: Acetylcholine; APACHE III: Acute physiology and chronic health evaluation III; ARDS: Acute respiratory distress syndrome; BAL: Bronchoalveolar lavage; CI: 95% confidence interval; CIP: Critical illness polyneuromyopathy; CMAP: Compound muscle action potential; CPAP: Continuous positive airway pressure; EMG: Electromyographic; ICU: Intensive care unit; IL: Interleukin; MOF: Multi-organ failure; NMBAs: Neuromuscular blocking agents; OR: Odds ratio; P:F ration: Arterial partial pressure of oxygen to inspired oxygen ratio; PEEP: Positive end-expiratory pressure; SAPS II: Simplified acute physiology score II; TOF: Train of four.

## Competing interests

The authors of this review have no competing interests to disclose.

## Authors’ contributions

DP developed the concept for the review, researched the topic and analyzed the available evidence, wrote the primary manuscript, and made multiple revisions to the manuscript. NK approved the concept for review, aided in acquisition of evidence, revised the manuscript to ensure intellectual content, and approved the final version for publication. NS revised the manuscript to ensure intellectual content, approved the final version for publication, and will serve as corresponding author. All authors read and approved the final manuscript.

## Authors’ information

DP is a Third-Year Resident in the Department of Internal Medicine with future plans for fellowship training in Pulmonary and Critical Care Medicine.

NK is an Associate Professor in the Division of Pulmonary, Critical Care, and Sleep Medicine. NK is Co-Director of UC Davis Asthma Network as well as Associate Program Director of the Pulmonary and Critical Care Fellowship Program. NK’s clinical interests focus on asthma, non-invasive markers of airway disease, chronic obstructive pulmonary disease and sepsis. NK’s research interests include airway inflammation and fibrosis, lung physiology, environmental effects on lung function, the role of nitric oxide in airway diseases, asthma, chronic obstructive pulmonary disease and lung injury.

NS is an Assistant Clinical Professor in the Division of Pulmonary, Critical Care, and Sleep Medicine. NS is a clinician-educator who’s clinical and research interests include bedside point-of-care ultrasound, interventional bronchoscopy, critical care medicine, and cystic fibrosis.
